# Growth Differentiation Factor 15 Is Associated with Severe Coronary Artery Calcification in Patients with Chronic Kidney Disease

**DOI:** 10.3390/life16101661

**Published:** 2026-10-02

**Authors:** Maria Kislikova, Paola Nova Montoya, Laura García García, José Antonio Parra Blanco, María Teresa García Unzueta, Vicente Celestino Pinera Haces, María de la Oliva Valentín Munoz, Adalberto Benito Hernández, Juan Carlos Ruiz San Millán, Emilio Rodrigo

**Affiliations:** 1 Immunopathology Group, Nephrology Department, Marqués de Valdecilla University Hospital-IDIVAL, University of Cantabria, 39008 Santander, Spain; paolaandrea.nova@scsalud.es (P.N.M.); laura_gtineo@hotmail.com.es (L.G.G.); vicentecelestino.pinera@scsalud.es (V.C.P.H.); mariadelaoliva.valentin@scsalud.es (M.d.l.O.V.M.); adalberto.benito@scsalud.es (A.B.H.); juancarlos.ruiz@scsalud.es (J.C.R.S.M.); 2 Radiology Department, Marqués de Valdecilla University Hospital-IDIVAL, University of Cantabria, 39008 Santander, Spain; parraja@unican.es; 3 Clinical Laboratory Department, Marqués de Valdecilla University Hospital-IDIVAL, 39008 Santander, Spain; mteresa.garciau@scsalud.es

**Keywords:** growth differentiation factor-15, chronic kidney disease, coronary artery calcification, cardiovascular events, matrix Gla protein

## Abstract

Patients with chronic kidney disease (CKD) have a high burden of coronary artery calcification (CAC) and cardiovascular risk. GDF-15 is a stress-responsive biomarker linked to kidney dysfunction and adverse cardiovascular outcomes, but its relationship with CAC across CKD stages is incompletely characterized. We evaluated associations of GDF-15 with severe CAC and major adverse cardiovascular events (MACE). In this prospective observational study, 128 patients with CKD and no previous cardiovascular disease had baseline GDF-15, dp-ucMGP, high-sensitivity cardiac troponin I, and NT-proBNP measured; CAC was quantified by computed tomography in 127. Severe CAC was defined as Agatston score ≥ 400. Mean serum GDF-15 was 4.5 ± 3.4 ng/mL. GDF-15 discriminated severe CAC (AUC 0.728; 95% CI 0.642–0.804; *p* < 0.001). Each doubling of GDF-15 was associated with higher odds of severe CAC after adjustment for eGFR, diabetes, LDL cholesterol, and phosphate (OR 2.105; 95% CI 1.045–4.240; *p* = 0.037), but this association was attenuated after adding high-sensitivity troponin I (*p* = 0.086). During 19 months, nine MACE occurred; each doubling of GDF-15 was associated with higher MACE risk (HR 2.679; 95% CI 1.278–5.625; *p* = 0.009). Higher GDF-15 was associated with severe CAC and cardiovascular events, supporting further evaluation in CKD.

## 1. Introduction

Chronic kidney disease (CKD) is a major global health problem and is associated with a markedly increased risk of cardiovascular morbidity and premature mortality. Cardiovascular disease is the leading cause of death among patients with CKD, and individuals with moderate-to-severe kidney dysfunction are frequently more likely to experience a cardiovascular event than to progress to kidney failure [1,2]. This excess risk cannot be fully explained by traditional cardiovascular risk factors, such as age, hypertension, diabetes mellitus, dyslipidemia, and smoking. CKD-specific mechanisms, including chronic inflammation, oxidative stress, endothelial dysfunction, disturbances in mineral and bone metabolism, and the accumulation of uremic toxins, also contribute to cardiovascular damage [3].

Coronary artery calcification (CAC) is highly prevalent in CKD, develops earlier, and progresses more rapidly than in individuals without kidney disease. Its prevalence and severity increase as renal function declines and are particularly high in patients with advanced CKD and those receiving dialysis [4,5]. CAC reflects the combined contribution of atherosclerotic intimal calcification and CKD-related medial vascular calcification. Disturbances in calcium and phosphate metabolism, secondary hyperparathyroidism, inflammation, oxidative stress, and an imbalance between promoters and inhibitors of calcification are involved in this process [4,6]. Importantly, CAC can be detected before the onset of clinically apparent cardiovascular disease and provides prognostic information regarding cardiovascular events and mortality. High Agatston scores, particularly values above 300–400 units, identify CKD patients at substantially increased cardiovascular risk [7].

The identification of circulating biomarkers associated with cardiovascular damage may improve risk stratification in CKD and provide insight into the biological pathways involved in vascular injury. Growth differentiation factor-15 (GDF-15) and matrix Gla protein (MGP) are of particular interest because they may act not only as biomarkers of cardiovascular risk and vascular calcification, but also as potential participants in their pathogenesis. GDF-15 is a stress-inducible cytokine belonging to the transforming growth factor-β superfamily. It is produced by several cell types, including cardiomyocytes, macrophages, vascular smooth muscle cells, and renal cells, in response to inflammation, oxidative stress, hypoxia, and tissue injury [8,9]. Circulating GDF-15 concentrations are frequently elevated in CKD and are strongly and inversely associated with estimated glomerular filtration rate [10,11]. Higher GDF-15 concentrations have been associated with CKD progression, incident heart failure, cardiovascular events, cardiovascular mortality, and all-cause mortality, including among patients undergoing dialysis [12,13,14,15]. A systematic review and dose–response meta-analysis showed that patients in the highest GDF-15 categories had approximately two- to threefold higher risks of cardiovascular events, cardiovascular mortality, and all-cause mortality than those in the lowest categories [15].

GDF-15 has also been associated with subclinical coronary atherosclerosis and CAC. In populations without overt cardiovascular disease, elevated GDF-15 concentrations were independently associated with the presence and severity of CAC, although this relationship may be modified by age and kidney function [16,17,18]. Nevertheless, evidence specifically addressing the relationship between GDF-15 and CAC across the full spectrum of CKD remains limited. MGP is a vitamin K-dependent inhibitor of vascular calcification, whereas its inactive dephosphorylated-uncarboxylated form, dp-ucMGP, reflects impaired MGP activation and has been proposed as a biomarker of vitamin K deficiency and vascular calcification risk in CKD [4].

We hypothesized that higher circulating GDF-15 concentrations would be independently associated with severe CAC and with an increased risk of major adverse cardiovascular events in patients across different stages of CKD, whereas dp-ucMGP would show a weaker association with coronary calcification. Accordingly, this study evaluated the relationships of GDF-15 and dp-ucMGP with CAC and prospectively assessed the association between GDF-15 and subsequent cardiovascular events.

## 2. Materials and Methods

### 2.1. Study Design and Participants

This prospective observational study included adult patients with chronic kidney disease (CKD) receiving follow-up in nephrology outpatient clinics, advanced CKD clinics, peritoneal dialysis units, or hemodialysis units. Kidney transplant recipients were not included. Participants were enrolled between 5 October 2020, and 31 January 2021. Eligible participants were aged ≥18 years, agreed to participate in the study, and provided written informed consent. Exclusion criteria were a history of cardiovascular disease, atrial fibrillation, and any previous diagnosis of malignancy, including hematologic malignancies. Inflammatory and autoimmune diseases were not exclusion criteria.

A total of 128 patients provided informed consent and were included in the study. GDF-15 and dp-ucMGP concentrations were measured in all 128 participants. A major cardiovascular event (MACE) was defined if the patients presented an acute myocardial infarction requiring PTCA/stent, coronary bypass, stroke, transient ischemic attack, peripheral arterial ischemia requiring PTCA/stent, amputations, limb bypass, arterial thrombosis, cardiogenic shock, or cardiac arrest. Coronary artery calcification was assessed in 127 participants because one patient died before undergoing computed tomography. Estimated glomerular filtration rate (eGFR) was calculated using the 2009 CKD-EPI creatinine equation. The calculated eGFR value was used in the statistical analyses across the study population, including participants receiving dialysis. In patients undergoing hemodialysis, blood samples were obtained before the first hemodialysis session of the week, following the long interdialytic interval. The study was approved by the Cantabria Clinical Research Ethics Committee (approval No. 2020.326), and all participants provided written informed consent.

### 2.2. Biomarker Measurement

Blood samples were collected at the baseline study visit. Aliquots of serum and plasma were prepared from each blood sample and stored at −80 °C until measurement. GDF-15 concentrations were measured in serum using the Human GDF-15 ELISA Kit (Invitrogen, Carlsbad, CA, USA), with an assay range of 1.1–800 pg/mL and a detection limit of 2 pg/mL. All serum samples were analyzed using both 1:5 and 1:15 dilutions. Results obtained with the two dilution factors were concordant across samples, including those with lower and higher GDF-15 concentrations. Final concentrations were calculated after correction for the corresponding dilution factor. Desphospho-uncarboxylated matrix Gla protein (dp-ucMGP) concentrations were measured in potassium EDTA plasma using the IDS-iSYS InaKtif MGP assay (Immunodiagnostic Systems Limited, Boldon, UK), with an assay range of 300–12,000 pmol/L and a detection limit of 300 pmol/L. High-sensitivity cardiac troponin I concentrations were measured in serum using the Atellica^®^ IM High-Sensitivity Troponin I assay (Siemens Healthcare Diagnostics Inc., Erlangen, Germany), with an assay range of 2.50–25,000 ng/L and a detection limit of 1.27 ng/L. NT-proBNP concentrations were measured in serum using the Atellica^®^ IM NT-proBNP assay (Siemens HealthCare, Malvern, PA, USA), with an assay range of 35–35,000 pg/mL and a detection limit of 20 pg/mL.

### 2.3. Assessment of Coronary Artery Calcification

Coronary artery calcification was assessed by non-contrast cardiac computed tomography using 64- or 128-detector scanners (Optima 64 and Revolution EVO 128, GE Healthcare, Chicago, IL, USA). Images were obtained using prospective acquisition with heart-rate monitoring. Coronary calcium was quantified using SmartScore software version 4.0 (GE Healthcare, USA) and expressed in Agatston units. Global coronary artery calcification was calculated as the total calcium score across the coronary arteries, and calcification of the left anterior descending coronary artery was also evaluated separately. For the primary analysis, participants were classified according to a global Agatston score < 400 or ≥400.

### 2.4. Statistical Analysis

Continuous variables with an approximately normal distribution were summarized as mean ± standard deviation and categorical variables were expressed as frequencies and percentages. Markedly skewed continuous variables were log2-transformed before parametric analyses. GDF-15, high-sensitivity cardiac troponin I, NT-proBNP, and PTH were analyzed after log2 transformation where indicated. GDF-15 concentrations were expressed in pg/mL before log2 transformation, high-sensitivity cardiac troponin I in ng/L, and NT-proBNP in pg/mL. Accordingly, for log2-transformed biomarkers, a one-unit increase corresponds to a doubling of the original biomarker concentration.

Variables associated with severe coronary artery calcification, defined as a global Agatston score ≥ 400, were first examined in univariable analyses. Continuous variables were compared between patients with an Agatston score < 400 and those with a score ≥ 400 using Student’s *t* test, whereas categorical variables were compared using the chi-square test or Fisher’s exact test, as appropriate. Multivariable binary logistic regression models were constructed using the enter method. Covariates were selected on the basis of clinical relevance, previous knowledge of determinants of coronary calcification in CKD, and findings from the univariable analyses, while maintaining parsimonious models because of the limited number of participants with severe CAC. The primary model included eGFR, diabetes mellitus, log2-transformed GDF-15, LDL cholesterol, and serum phosphate. Results were expressed as odds ratios and 95% confidence intervals.

To assess the robustness of the association between GDF-15 and severe CAC, a sensitivity analysis additionally included age and sex as clinically relevant potential confounders irrespective of their univariable *p* values. A further sensitivity analysis was restricted to participants not receiving dialysis. Additional models examined the effect of including log2-transformed high-sensitivity cardiac troponin I in the primary model, with and without further adjustment for age and sex. These extended models were considered sensitivity analyses because of the limited number of participants with severe CAC.

Multicollinearity among covariates included in the multivariable models was assessed using tolerance and variance inflation factors (VIF). A VIF > 5 was considered indicative of potentially relevant multicollinearity.

Receiver operating characteristic (ROC) curve analysis was used to assess the ability of GDF-15, dp-ucMGP, and high-sensitivity cardiac troponin I to discriminate between patients with an Agatston score < 400 and those with an Agatston score ≥ 400. Areas under the ROC curve (AUCs) and their 95% confidence intervals were calculated. Paired ROC curves obtained from the same participants were compared using the DeLong method. Individual AUC 95% confidence intervals were calculated using the exact binomial method. Formal paired comparisons were performed between GDF-15 and dp-ucMGP and between GDF-15 and high-sensitivity cardiac troponin I.

Variables associated with GDF-15 concentrations were also evaluated. Pearson correlation coefficients were used to assess associations between log2-transformed GDF-15 and continuous clinical and biochemical variables. Differences in log2-transformed GDF-15 concentrations between two independent groups were assessed using Student’s *t* test. Variables considered clinically relevant and associated with GDF-15 in the univariable analyses were subsequently included in a multivariable linear regression model. Highly correlated or conceptually redundant variables were not entered simultaneously into the same model.

Associations between clinical, biochemical, and imaging variables and the occurrence of major adverse cardiovascular events were evaluated using univariable Cox proportional hazards regression. Results were expressed as hazard ratios and 95% confidence intervals. Kaplan–Meier curves were constructed according to GDF-15 quartiles and compared using the log-rank test. Because only nine cardiovascular events occurred, multivariable Cox regression was not performed.

GDF-15 was considered the biomarker of primary interest. In an additional exploratory analysis, log2-transformed GDF-15, high-sensitivity cardiac troponin I, and NT-proBNP were entered simultaneously into a binary logistic regression model using the enter method to examine their respective associations with severe CAC.

Statistical analyses were performed using SPSS version 15.0 (SPSS Inc., Chicago, IL, USA), except for ROC curve analyses and paired ROC curve comparisons, which were performed using MedCalc version 23.7.1 (MedCalc Software Ltd., Ostend, Belgium). All tests were two-sided, and a *p* value < 0.05 was considered statistically significant.

## 3. Results

### 3.1. Study Population

GDF-15 and dp-ucMGP concentrations were measured in 128 patients, with a mean age of 61 ± 7 years. Coronary artery calcification was assessed in 127 patients because one participant died before undergoing the radiological examination. The characteristics of the study population are summarized in Table 1. The mean GDF-15 concentration was 4.5 ± 3.4 ng/mL, and the mean dp-ucMGP concentration was 1661 ± 1249 pmol/L. Among the 40 patients with stage 5 CKD, 28 were receiving hemodialysis, 4 were receiving peritoneal dialysis, and 8 were not yet receiving dialysis.

### 3.2. Relationship of GDF-15 and dp-ucMGP with Coronary Artery Calcification

Table 2 shows the variables associated with significant global coronary artery calcification, defined as an Agatston score ≥ 400. In the univariable analysis, patients with an Agatston score ≥ 400 had a higher prevalence of stage 5 CKD and diabetes mellitus, lower eGFR and LDL cholesterol levels, and higher concentrations of high-sensitivity cardiac troponin I, NT-proBNP, GDF-15, and serum phosphate.

**Figure 1:**
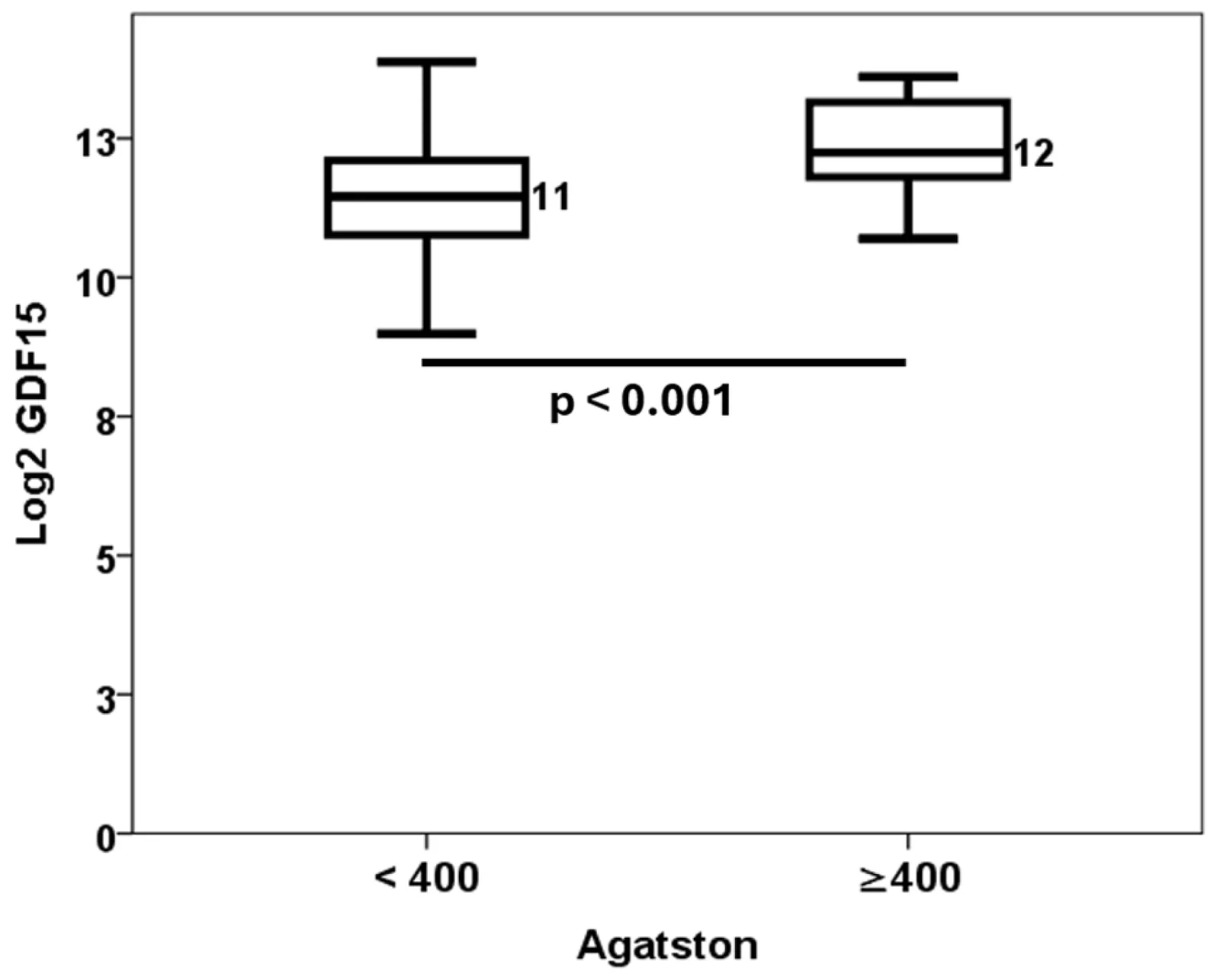
Box-and-whisker plot of Log2-transformed GDF-15 concentrations in patients with and without an Agatston score ≥ 400 (*p* < 0.001).

**Figure 2:**
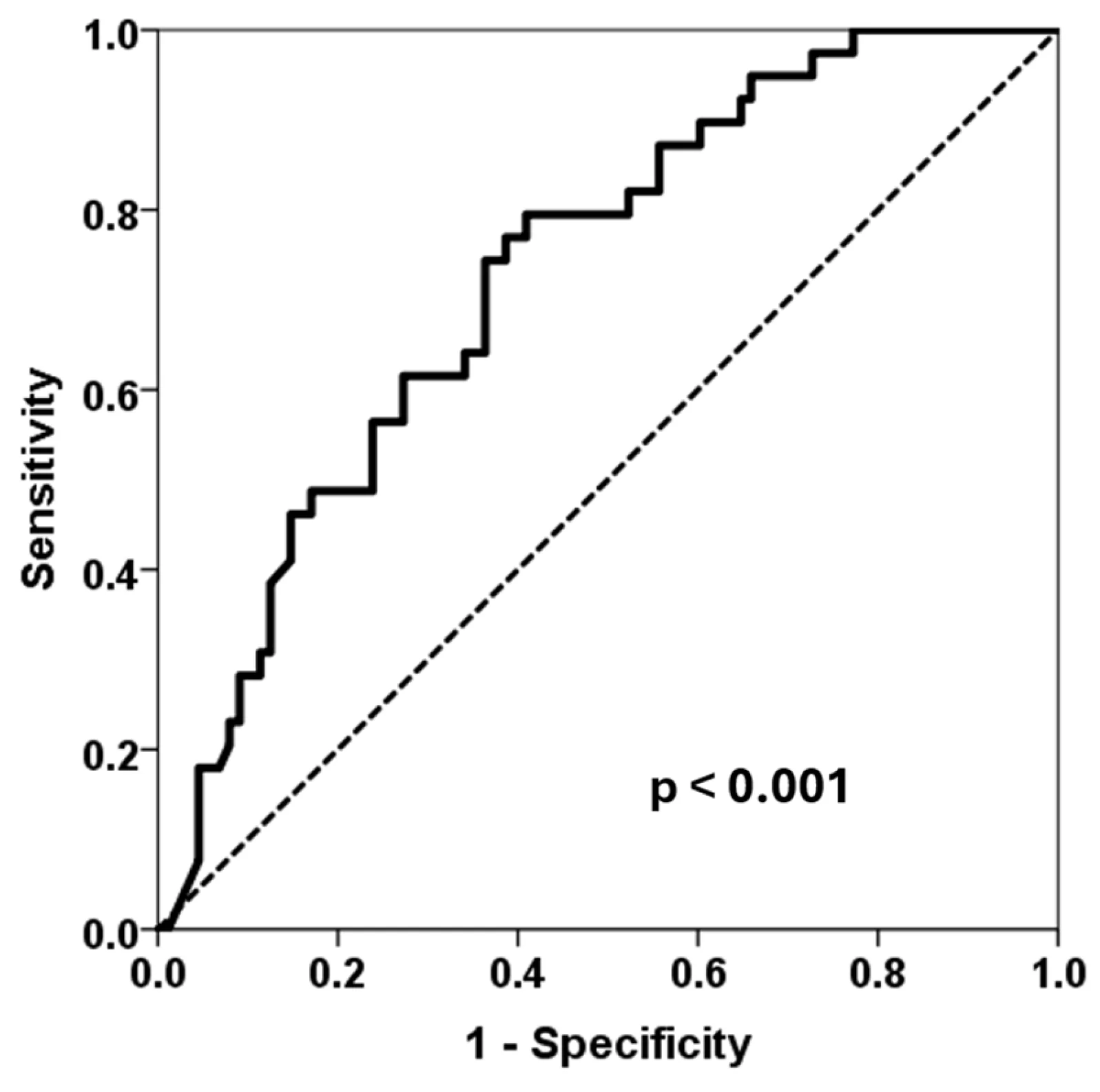
Receiver operating characteristic curve for GDF-15 discrimination of patients with an Agatston score ≥ 400 (AUC, 0.728; 95% CI, 0.642–0.804).

Log2-transformed GDF-15 was significantly higher in patients with an Agatston score ≥ 400 than in those with an Agatston score < 400 (Figure 1). GDF-15 showed significant discrimination for an Agatston score ≥ 400, with an area under the receiver operating characteristic curve (AUC) of 0.728 (95% CI, 0.642–0.804, *p* < 0.001; Figure 2). The corresponding AUC for dp-ucMGP was 0.610 (95% CI, 0.519–0.695, *p* = 0.056). In a formal paired comparison using the DeLong method, the AUC for GDF-15 was significantly higher than that for dp-ucMGP (difference between AUCs, 0.118; 95% CI, 0.007–0.230; *p* = 0.037). The discriminatory performance of GDF-15 was similar to that of high-sensitivity cardiac troponin I (AUC, 0.728; 95% CI, 0.642–0.804 vs. 0.726; 95% CI, 0.640–0.801, respectively), with no significant difference between the paired ROC curves (difference between AUCs, 0.002; 95% CI, −0.100 to 0.105; *p* = 0.964).

In an exploratory model including log2-transformed GDF-15, log2-transformed high-sensitivity cardiac troponin I, and log2-transformed NT-proBNP simultaneously, GDF-15 (OR, 1.875; 95% CI, 1.214–2.897; *p* = 0.005) and high-sensitivity cardiac troponin I (OR, 1.476; 95% CI, 1.065–2.045; *p* = 0.019) were associated with an Agatston score ≥ 400, whereas NT-proBNP was not (OR, 0.927; 95% CI, 0.725–1.186; *p* = 0.547).

The primary multivariable logistic regression model included eGFR, diabetes mellitus, log2-transformed GDF-15, LDL cholesterol, and serum phosphate (n = 127) (Table 3). Diabetes mellitus (OR, 5.560; 95% CI, 2.110–14.651; *p* = 0.001), serum phosphate (OR per 1-mg/dL increase, 1.558; 95% CI, 1.040–2.333; *p* = 0.032), and GDF-15 were independently associated with an Agatston score ≥ 400. Each doubling of GDF-15 concentration was associated with approximately twofold higher odds of severe CAC (OR, 2.105; 95% CI, 1.045–4.240; *p* = 0.037). Neither eGFR (OR, 1.009; 95% CI, 0.984–1.035; *p* = 0.478) nor LDL cholesterol (OR, 0.997; 95% CI, 0.983–1.010; *p* = 0.621) was independently associated with an Agatston score ≥ 400.

In a sensitivity analysis additionally including age and sex as clinically relevant potential confounders, the magnitude of the association between GDF-15 and severe CAC was only minimally attenuated (Table 3). Each doubling of GDF-15 was associated with an OR of 2.008 (95% CI, 0.976–4.131; *p* = 0.058), compared with an OR of 2.105 in the primary model. Neither age (OR, 1.026; 95% CI, 0.955–1.102; *p* = 0.480) nor sex (OR, 1.679; 95% CI, 0.603–4.672; *p* = 0.321) was independently associated with severe CAC. In this extended model, diabetes mellitus (OR, 4.548; 95% CI, 1.649–12.542; *p* = 0.003) and serum phosphate (OR, 1.682; 95% CI, 1.081–2.617; *p* = 0.021) remained associated with an Agatston score ≥ 400, whereas eGFR (OR, 1.010; 95% CI, 0.984–1.036; *p* = 0.461) and LDL cholesterol (OR, 0.998; 95% CI, 0.984–1.012; *p* = 0.757) were not.

Assessment of multicollinearity showed no evidence of severe collinearity among the covariates. The highest VIF values were observed for log2-transformed GDF-15 (VIF, 3.31; tolerance, 0.302) and eGFR (VIF, 3.12; tolerance, 0.320), consistent with their strong correlation, whereas the remaining covariates had substantially lower VIF values.

A further sensitivity analysis was restricted to participants who were not receiving dialysis (n = 95) (Supplementary Table S1). In this subgroup, the association between GDF-15 and severe CAC remained in the same direction and was of similar or greater magnitude than in the overall cohort. Each doubling of GDF-15 was associated with an OR of 2.597 (95% CI, 0.932–7.233; *p* = 0.068) for an Agatston score ≥ 400. After additional adjustment for age and sex, the corresponding OR was 3.066 (95% CI, 0.975–9.637; *p* = 0.055). The wider confidence intervals in these analyses indicate lower precision after restriction to the smaller non-dialysis subgroup.

When log2-transformed high-sensitivity cardiac troponin I was added to the primary multivariable model, the point estimate for GDF-15 was only modestly attenuated, although the confidence interval widened and the association no longer reached conventional statistical significance (OR, 1.879; 95% CI, 0.915–3.859; *p* = 0.086) (Table 3). High-sensitivity cardiac troponin I was associated with severe CAC in this model (OR, 1.515; 95% CI, 1.045–2.195; *p* = 0.028). Diabetes mellitus (OR, 5.596; 95% CI, 2.048–15.291; *p* = 0.001) and serum phosphate (OR, 1.522; 95% CI, 1.005–2.304; *p* = 0.047) also remained associated with severe CAC, whereas eGFR (OR, 1.014; 95% CI, 0.988–1.040; *p* = 0.295) and LDL cholesterol (OR, 0.997; 95% CI, 0.983–1.011; *p* = 0.673) were not. Further adjustment for age and sex produced very similar estimates for GDF-15 (OR, 1.834; 95% CI, 0.876–3.836; *p* = 0.107) and high-sensitivity cardiac troponin I (OR, 1.472; 95% CI, 1.009–2.148; *p* = 0.045), whereas neither age (OR, 1.015; 95% CI, 0.945–1.091; *p* = 0.680) nor sex (OR, 1.429; 95% CI, 0.499–4.096; *p* = 0.506) was independently associated with severe CAC.

The primary model included eGFR, diabetes mellitus, log2-transformed GDF-15, LDL cholesterol, and serum phosphate. Sensitivity models additionally included age and sex or log2-transformed high-sensitivity cardiac troponin I (hs-cTnI). For log2-transformed biomarkers, the odds ratio corresponds to a doubling of the original biomarker concentration.

### 3.3. Clinical and Biochemical Variables Associated with GDF-15

The correlations between GDF-15 concentrations and continuous clinical and biochemical variables are shown in Table 4. GDF-15 was positively correlated with high-sensitivity troponin I, NT-proBNP, serum phosphate, parathyroid hormone, left anterior descending artery calcification, and global coronary artery calcification. Conversely, GDF-15 was inversely correlated with eGFR, LDL cholesterol and serum calcium. Log2-transformed GDF-15 concentrations were significantly higher in diabetic patients (12.1 ± 0.8 vs. 11.5 ± 1.2, *p* = 0.001), but did not differ significantly between men and women (11.8 ± 1.1 vs. 11.7 ± 1.2, respectively; *p* = 0.639) and between smokers and nonsmokers (11.7 ± 1.1 vs. 11.7 ± 1.1, respectively; *p* = 0.834).

**Figure 3:**
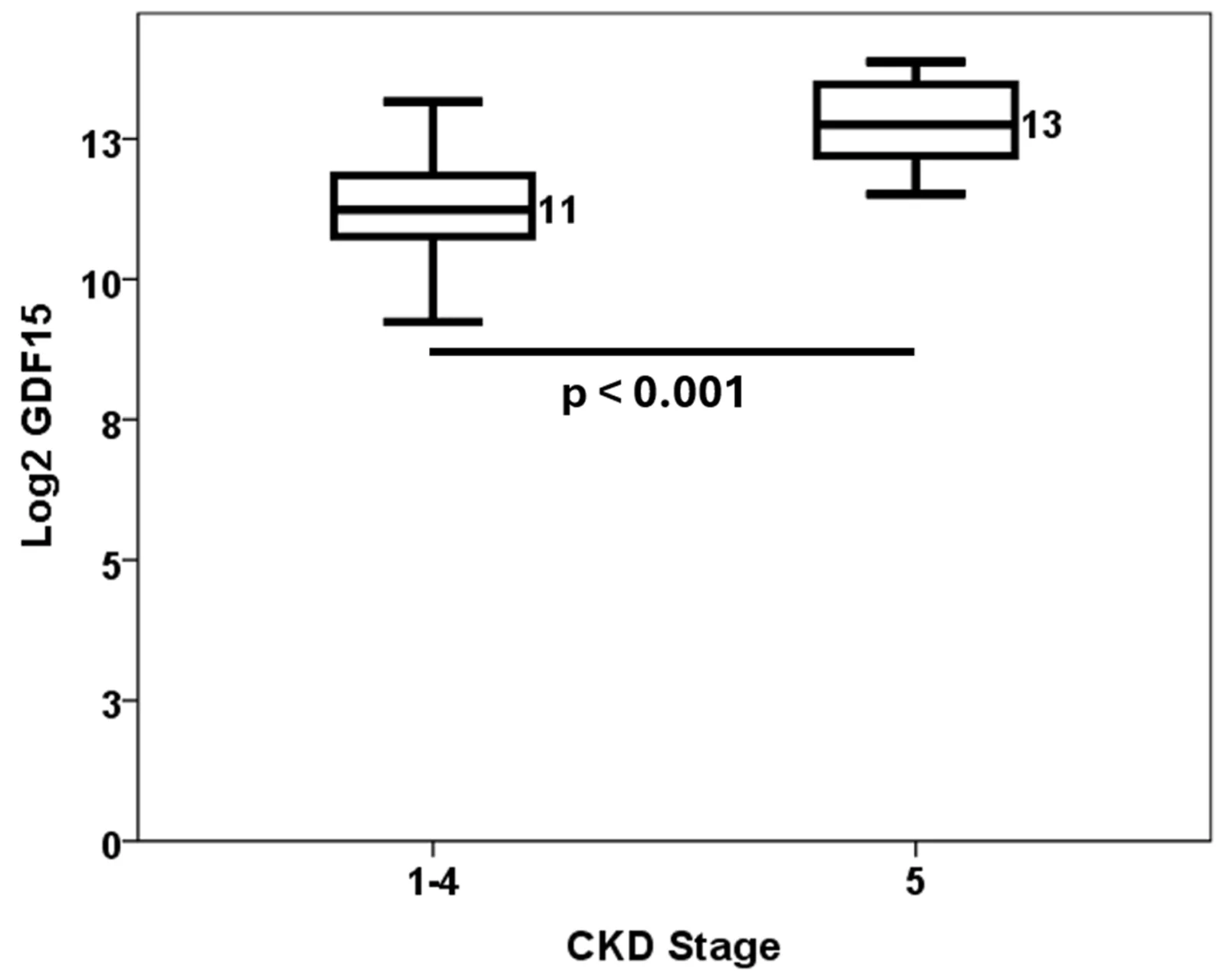
Box-and-whisker plot of Log_2_ GDF-15 concentrations in patients with and without stage 5 CKD (*p* < 0.001).

Patients with stage 5 CKD had significantly higher Log2-transformed GDF-15 than patients with CKD stages 1–4 (12.8 ± 0.7 vs. 11.2 ± 1.0, *p* < 0.001; Figure 3).

**Figure 4:**
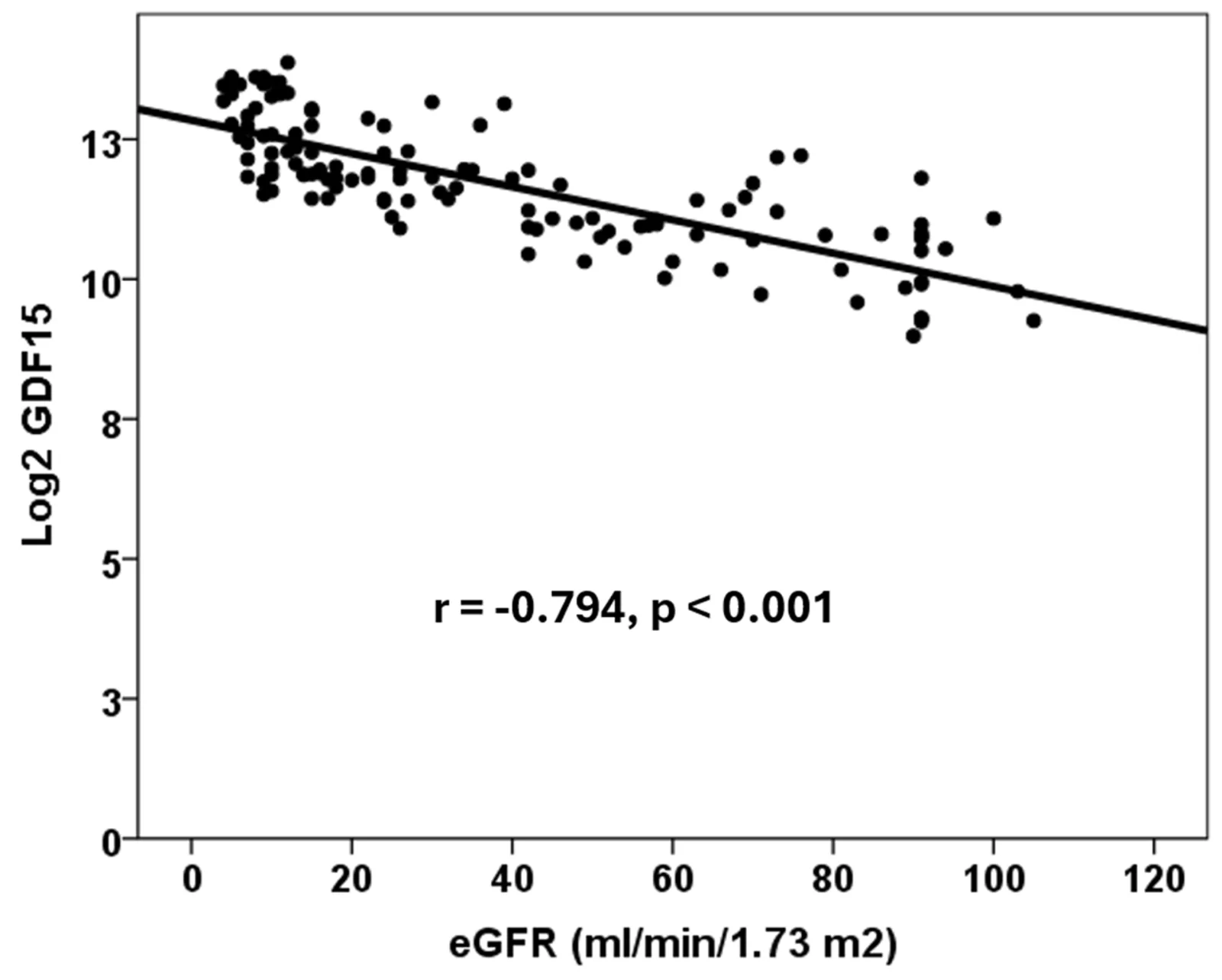
Correlation between log2-transformed GDF-15 and eGFR (r = −0.794, *p* < 0.001).

Variables associated with GDF-15 concentrations were subsequently examined using multivariable linear regression analysis (Table 4). eGFR, NT-proBNP and diabetes mellitus were independently associated with higher GDF-15 concentrations. The inverse correlation between log2-transformed GDF-15 and eGFR is shown in Figure 4 (r = −0.794; *p* < 0.001).

### 3.4. GDF-15 and Cardiovascular Events

During a median follow-up of 19 months, nine patients experienced a major adverse cardiovascular event: five acute myocardial infarctions requiring PTCA/stent or coronary bypass, one transient ischemic attack, two peripheral arterial ischemic events requiring PTCA/stent, and one cardiac arrest. Five events occurred among the 32 patients in the highest GDF-15 quartile (15.6%), compared with four events among the 96 patients in the three lower quartiles (4.2%).

The results of the univariable Cox regression analyses are presented in Table 5. Higher GDF-15 concentrations were associated with an increased risk of MACE. Each doubling of GDF-15 concentration was associated with a 2.68-fold higher risk of MACE (HR, 2.679; 95% CI, 1.278–5.625; *p* = 0.009). Stage 5 CKD, lower eGFR, higher NT-proBNP, lower HDL cholesterol, higher serum phosphate, higher parathyroid hormone, and greater coronary artery calcification were also significantly associated with MACE in the univariable analyses. An Agatston score ≥ 400 was associated with an increased risk of MACE (HR, 8.223; 95% CI, 1.012–66.842; *p* = 0.049).

**Figure 5:**
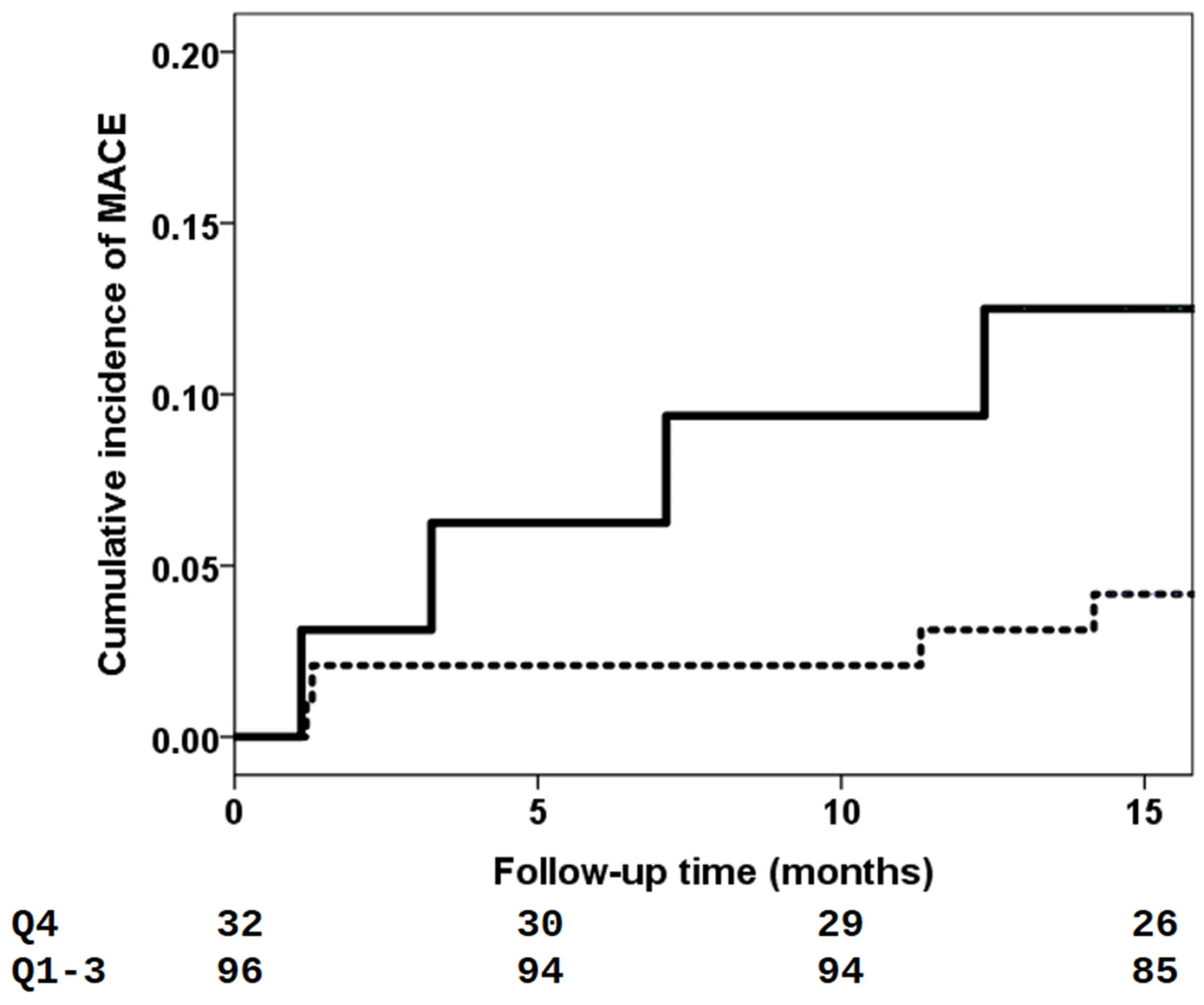
Kaplan–Meier curve for MACE comparing patients in the highest GDF-15 quartile (solid line) with those in the three lower quartiles (dashed line; log-rank statistic, 5.019; *p* = 0.025). Numbers at risk are shown at 0, 5, 10 and 15 months. Q4 = fourth quartile; Q1–3 = quartiles 1–3.

Kaplan–Meier analysis showed a significantly higher cumulative incidence of MACE among patients in the highest GDF-15 quartile than among those in the three lower quartiles (Figure 5; 12-month cumulative incidence, 12.5% vs. 3.1%; log-rank *p* = 0.025). Because only nine cardiovascular events occurred, multivariable Cox regression was not performed. These prognostic findings should therefore be considered exploratory.

## 4. Discussion

### 4.1. Prevalence of Severe Coronary Artery Calcification Across CKD Stages

Severe coronary artery calcification was frequent in our cohort, particularly among the 39 patients with stage 5 CKD, in whom 17 (43.6%) had an Agatston score ≥ 400. Previous studies consistently demonstrate a substantial burden of coronary and vascular calcification across CKD, with prevalence and severity increasing in more advanced kidney disease. In CRIC, CAC was present in 65% of participants with CKD stages 2–4 [19], while a meta-analysis of CKD cohorts reported an overall CAC prevalence of 60% (95% CI, 53–68%), with a pooled prevalence of 65% among patients receiving hemodialysis [20]. Advanced CKD cohorts have similarly shown a high frequency of extensive arterial calcification, including calcium scores > 400 [21]. Direct numerical comparison with our findings should be made cautiously because previous studies differ considerably in CKD stage distribution, dialysis exposure, diabetes prevalence, age, vascular territory assessed, and CAC thresholds. Overall, our findings are consistent with the marked calcific burden characteristic of advanced CKD.

### 4.2. Clinical Relevance of Coronary Artery Calcification in CKD

The high prevalence of CAC in CKD is clinically relevant because coronary calcium is not merely an imaging finding but a marker of adverse cardiovascular prognosis. A meta-analysis of 38 studies reported substantially higher risks of all-cause mortality (HR 3.44, 95% CI 2.40–4.94), cardiovascular mortality (HR 3.87, 95% CI 2.06–7.26), and cardiovascular events (HR 2.09, 95% CI 1.19–3.67) among patients in the highest CAC category [20]. Similarly, in CRIC, each 1-SD increase in log-transformed CAC was independently associated with cardiovascular disease (HR 1.40, 95% CI 1.16–1.69), myocardial infarction (HR 1.44, 95% CI 1.02–2.02), and heart failure (HR 1.39, 95% CI 1.10–1.76) after adjustment for traditional and CKD-related risk factors [22]. Longitudinal data from the CRIC study showed that incident CAC was associated with a 2.42-fold higher risk of atherosclerotic cardiovascular disease and a 1.82-fold higher risk of all-cause mortality, while CAC progression was associated with a 1.73-fold higher risk of all-cause mortality [23]. These observations provide the clinical context for our use of an Agatston score ≥ 400 as a marker of severe coronary calcification and high cardiovascular risk.

### 4.3. Association Between GDF-15 and Severe Coronary Artery Calcification

The main finding of our study is the association between higher circulating GDF-15 concentrations and severe CAC. GDF-15 concentrations were higher in patients with an Agatston score ≥ 400, and GDF-15 showed significant discriminatory ability for severe CAC, with an AUC of 0.728 (95% CI, 0.642–0.804; *p* < 0.001). This observation is consistent with previous evidence linking GDF-15 to subclinical coronary atherosclerosis and calcification. In the Dallas Heart Study, GDF-15 ≥ 1800 ng/L was independently associated with both CAC > 10 (OR 2.1, 95% CI 1.2–3.7) and CAC ≥ 100 (OR 2.6, 95% CI 1.4–4.9) after adjustment for traditional risk factors, renal function, and left ventricular mass [16]. In advanced kidney disease, GDF-15 has also been associated with greater vascular calcification and inflammatory activity [24].

The magnitude of GDF-15 elevation in our cohort is also consistent with the advanced renal disease represented in the study. Mean GDF-15 was 4.5 ± 3.4 ng/mL, markedly higher than concentrations generally observed in community-based populations and similar to those reported in advanced CKD and end-stage kidney disease. In the Dallas Heart Study, GDF-15 concentrations ≥ 1.8 ng/mL already identified the highest prespecified category in a community-based population [16], whereas in patients with ESKD receiving hemodialysis, median GDF-15 concentrations reached 5.48 ng/mL (interquartile range, 3.96–7.53 ng/mL) [25]. The higher GDF-15 concentrations observed in advanced kidney dysfunction are biologically plausible because GDF-15 is a stress-responsive cytokine whose expression is induced by inflammation, oxidative stress, hypoxia, and other forms of cellular and tissue injury [8].

Of interest, the association with severe CAC was not explained solely by conventional clinical and biochemical risk factors. In the multivariable model including eGFR, diabetes, LDL cholesterol, serum phosphate, and GDF-15, each doubling of GDF-15 was associated with approximately twofold higher odds of an Agatston score ≥ 400 (OR 2.105, 95% CI 1.045–4.240; *p* = 0.037). This finding supports an association between GDF-15 and severe CAC after adjustment for several major clinical and biochemical determinants of calcification in CKD. However, because creatinine-based eGFR has limited validity in patients receiving dialysis, residual confounding by kidney function cannot be excluded and the adjusted association should be interpreted cautiously.

The relationship with cardiac biomarkers deserves a more nuanced interpretation. When GDF-15, high-sensitivity cardiac troponin I, and NT-proBNP were evaluated simultaneously, both GDF-15 (OR 1.875, 95% CI 1.214–2.897; *p* = 0.005) and high-sensitivity cardiac troponin I (OR 1.476, 95% CI 1.065–2.045; *p* = 0.019) remained associated with severe CAC. However, when high-sensitivity troponin I was subsequently added to the clinical multivariable model, the association of GDF-15 was attenuated and no longer reached conventional statistical significance (OR 1.879, 95% CI 0.915–3.859; *p* = 0.086), whereas troponin remained significant. The point estimate for GDF-15, however, changed little after troponin was added and remained in the same direction. The loss of statistical significance may partly reflect the correlation between these biomarkers and the reduced precision of a model including several related variables. For this reason, the result does not necessarily indicate that the association disappeared. It also does not allow us to conclude that troponin mediates the relationship between GDF-15 and CAC.

GDF-15 and cardiac troponin probably reflect different, although partly overlapping, aspects of cardiovascular disease in advanced CKD. GDF-15 is a broad stress-responsive biomarker linked to inflammation, oxidative stress, tissue injury, renal dysfunction, and adverse cardiovascular outcomes [8,10], whereas cardiac troponin more directly reflects myocardial injury and may remain chronically elevated in patients with CKD even in the absence of acute myocardial infarction [2,26]. Severe CAC may occur in patients who also have systemic cardiorenal stress and chronic myocardial injury. This could explain, at least in part, why GDF-15 and high-sensitivity cardiac troponin I provide overlapping information. Our results support an association between GDF-15 and severe CAC, although it remains unclear whether GDF-15 adds information beyond high-sensitivity cardiac troponin I. Our study was not designed to assess mediation or to establish causal relationships.

### 4.4. Clinical and Biochemical Determinants of GDF-15

Kidney function showed the strongest relationship with GDF-15 in our cohort. Log2-transformed GDF-15 was closely and inversely correlated with eGFR (r = −0.794; *p* < 0.001), and eGFR remained independently associated with GDF-15 in multivariable linear regression. Patients with stage 5 CKD also had markedly higher Log2-transformed GDF-15 concentrations than those with stages 1–4 (12.8 ± 0.7 vs. 11.2 ± 1.0; *p* < 0.001). These findings are consistent with previous CKD studies showing a strong inverse relationship between GDF-15 and kidney function and an association with subsequent CKD progression. In two independent CKD cohorts, each doubling of GDF-15 was associated with a 72% and 65% higher risk of kidney disease progression, respectively [10]. These findings underscore the importance of considering kidney function when interpreting circulating GDF-15 concentrations in patients with CKD.

Diabetes was another independent determinant of GDF-15 in our study, with higher Log2-transformed GDF-15 concentrations in patients with diabetes (12.1 ± 0.8 vs. 11.5 ± 1.2; *p* = 0.001). This finding is consistent with previous studies showing higher circulating GDF-15 concentrations in patients with type 2 diabetes and with evidence linking GDF-15 to insulin resistance and broader cardiometabolic stress [27,28]. By contrast, GDF-15 concentrations did not differ between men and women (11.8 ± 1.1 vs. 11.7 ± 1.2; *p* = 0.639) or between smokers and nonsmokers (11.7 ± 1.1 in both groups; *p* = 0.834). Sex-specific associations of GDF-15 have been reported in selected populations with atherosclerotic disease and ESKD, although the patterns observed have differed according to population and outcome [24,29]. In our cohort, kidney dysfunction and diabetes showed the strongest clinical relationships with GDF-15.

### 4.5. Other Variables Associated with Severe Coronary Artery Calcification

The clinical profile associated with severe CAC in our cohort was also consistent with previously described determinants of coronary calcification in CKD. Patients with an Agatston score ≥ 400 had lower eGFR in univariable analysis (*p* = 0.008), although eGFR did not remain independently associated after multivariable adjustment. Previous CKD studies have similarly shown that reduced kidney function is associated with greater CAC burden and progression [19,30]. Diabetes showed a stronger relationship in our cohort: it was more prevalent among patients with severe CAC and remained independently associated with an Agatston score ≥ 400 (OR 5.560, 95% CI 2.110–14.651; *p* = 0.001). This is consistent with longitudinal data showing greater CAC severity and progression in patients with CKD and diabetes than in those without diabetes [31]. Serum phosphate was also independently associated with severe CAC (OR 1.558 per 1-mg/dL increase, 95% CI 1.040–2.333; *p* = 0.032), in agreement with previous studies identifying higher serum phosphate as an independent determinant of CAC progression in CKD [19,31]. In contrast, although LDL cholesterol was lower among participants with severe CAC in univariable analysis, LDL cholesterol was not independently associated in the multivariable model. The inverse unadjusted association should not be interpreted as protective and may, at least in part, reflect lipid-lowering treatment and residual confounding.

### 4.6. Potential Explanation for the Association Between GDF-15 and Coronary Calcification

The present study demonstrates an association between GDF-15 and severe CAC but cannot establish that GDF-15 directly promotes vascular calcification. GDF-15 is a stress-responsive cytokine expressed by several cardiovascular and non-cardiovascular cell types in response to inflammation, oxidative stress, hypoxia, ischemia, and other forms of cellular injury [8]. These processes are particularly relevant in CKD, where chronic inflammation and oxidative stress coexist with disturbances in calcium–phosphate metabolism, endothelial dysfunction, and accelerated vascular ageing, all of which contribute to cardiovascular injury and vascular calcification [32,33]. GDF-15 may therefore reflect several biological processes occurring in CKD and may not be specific to vascular calcification.

This interpretation is supported by previous observations. In the Dallas Heart Study, the association between GDF-15 and CAC remained significant after extensive adjustment [16]. In end-stage kidney disease, higher GDF-15 has been linked to greater vascular calcification and IL-6 [24]. Other cardiovascular cohorts have shown that GDF-15 provides information related to broader inflammatory and tissue-remodeling processes and adds prognostic information for adverse cardiovascular outcomes [34,35]. Together with the attenuation seen after troponin was added to the fully adjusted model, these findings suggest that GDF-15 may reflect the broader cardiovascular and systemic stress associated with severe CAC. Further mechanistic studies will be needed to clarify whether GDF-15 has a direct role in vascular calcification or mainly reflects the underlying disease.

### 4.7. GDF-15 and Major Adverse Cardiovascular Events

Higher GDF-15 was also associated with subsequent cardiovascular events. Each doubling of GDF-15 was associated with a 2.68-fold higher risk of MACE (HR 2.679, 95% CI 1.278–5.625; *p* = 0.009), and patients in the highest GDF-15 quartile had a higher 12-month cumulative incidence of MACE than those in the lower three quartiles (12.5% vs. 3.1%; log-rank *p* = 0.025). This direction is consistent with the broad literature linking elevated GDF-15 to adverse cardiovascular outcomes. In an individual-patient meta-analysis of 53,486 patients with atherosclerotic cardiovascular disease, 3-year cumulative incidence of MACE increased stepwise from 7.8% at GDF-15 < 1200 ng/L to 11.4% at 1200–1800 ng/L and 16.8% at >1800 ng/L [35]. In patients with end-stage kidney disease receiving hemodialysis, each doubling of GDF-15 was independently associated with MACE (SHR 1.47, 95% CI 1.11–1.94) and all-cause mortality (SHR 1.58, 95% CI 1.28–1.95) [25].

Given the small number of events, the prognostic findings should be interpreted cautiously. Only nine MACE occurred during follow-up, resulting in wide confidence intervals and precluding reliable multivariable Cox regression. Consequently, we cannot determine whether the association between GDF-15 and MACE is independent of kidney function, CAC burden, troponin, NT-proBNP, or other cardiovascular risk factors. Our findings are consistent with a prognostic role for GDF-15, although this will need to be confirmed in larger CKD cohorts with more cardiovascular events.

### 4.8. dp-ucMGP and Coronary Artery Calcification

In contrast to GDF-15, dp-ucMGP concentrations did not differ significantly between patients with and without an Agatston score ≥ 400, and its discriminatory performance was modest (AUC 0.610). The weak association observed in our study does not contradict the known biological role of MGP in vascular calcification. MGP is a vitamin K-dependent inhibitor of vascular calcification that requires vitamin K-dependent carboxylation and phosphorylation to become biologically active, whereas dephosphorylated-uncarboxylated MGP (dp-ucMGP) represents an inactive form and is widely used as a circulating marker of poor vitamin K status [36,37]. Published associations between circulating MGP and vascular calcification have been heterogeneous, depending on the MGP fraction measured, the characteristics of the study population, and whether cross-sectional calcification burden or longitudinal progression was assessed [38]. More recently, higher dp-ucMGP concentrations were associated with long-term incidence and progression of coronary and thoracic aortic calcification in MESA [39], whereas studies in advanced CKD have also linked higher dp-ucMGP concentrations to greater vascular calcification burden [40]. In our cohort, dp-ucMGP showed a weaker relationship with severe CAC than GDF-15. This finding does not exclude a role for the vitamin K-MGP pathway in vascular calcification.

### 4.9. Limitations

Several limitations should be considered. First, the sample size was modest and the number of patients with severe CAC, as well as the number of cardiovascular events, was limited. This reduces precision in multivariable models and is particularly relevant when correlated biomarkers such as GDF-15 and troponin are entered simultaneously. Second, the cross-sectional relationship between baseline biomarkers and CAC does not establish temporality or causality, and a single CAC assessment cannot determine whether GDF-15 predicts calcification progression. Third, the cohort included patients across a broad spectrum of CKD, including advanced CKD and dialysis, which increases clinical heterogeneity and makes kidney function an important potential confounder when interpreting circulating GDF-15. In addition, creatinine-based eGFR equations have limited validity for estimating residual kidney function in patients receiving dialysis. Therefore, adjustment for eGFR in analyses including dialysis patients may not fully account for differences in kidney function, and residual confounding cannot be excluded. Fourth, several cardiovascular and renal biomarkers were evaluated and residual confounding cannot be excluded. Fifth, inflammatory and autoimmune diseases were not exclusion criteria, and concomitant medications were not systematically analyzed as potential determinants of GDF-15 concentrations; therefore, residual confounding related to inflammatory activity or drug-related effects on circulating GDF-15 cannot be excluded. Finally, the findings derive from a single cohort and require external validation.

## 5. Conclusions

In patients with CKD, higher circulating GDF-15 concentrations were associated with severe coronary artery calcification and showed better discrimination of an Agatston score ≥ 400 than dp-ucMGP. The association between GDF-15 and severe CAC persisted after adjustment for major clinical and biochemical determinants of calcification, including eGFR, diabetes, LDL cholesterol, and serum phosphate, although this adjusted association should be interpreted cautiously because creatinine-based eGFR is of limited validity in patients receiving dialysis. Additional adjustment for high-sensitivity cardiac troponin I attenuated the statistical significance of the association without materially changing its direction or magnitude, suggesting partially overlapping cardiovascular information rather than demonstrating absence of an association. GDF-15 was also associated with subsequent MACE, although the small number of events precludes conclusions regarding independent prognostic value. Overall, these findings suggest that GDF-15 may be a promising marker of cardiovascular disease burden in CKD and support further evaluation in larger longitudinal cohorts before its incremental clinical value can be established.

## 6. Future Directions

Future studies should validate these findings in larger, stage-stratified CKD cohorts with serial assessment of GDF-15 and CAC and sufficient cardiovascular events to determine whether GDF-15 predicts CAC progression and provides incremental prognostic information beyond kidney function, CAC burden, high-sensitivity cardiac troponin I, and NT-proBNP. Mechanistic studies are also needed to clarify whether GDF-15 participates directly in vascular calcification or predominantly reflects broader cardiorenal and systemic stress. From a clinical perspective, the present findings are insufficient to support the routine use of GDF-15 for cardiovascular risk stratification in CKD. However, if its incremental value is confirmed, GDF-15 could potentially complement established clinical, biochemical, and imaging markers in identifying patients with a high burden of coronary calcification or cardiovascular risk.

## Figures and Tables

**Table 1:** Characteristics of the study population.

	Overall
Age (years)	61 ± 7
Male sex	70 (54.7%)
BMI (kg/m^2^)	28.3 ± 5.3
CKD stage	
1	16 (12.5%)
2	17 (13.3%)
3	28 (21.9%)
4	27 (21.1%)
5	40 (31.3%)
eGFR (mL/min/1.73 m^2^)	38.1 ± 30.4
CKD etiology	
*Glomerular*	29 (22.7%)
*Diabetic*	22 (17.2%)
*Vascular*	19 (14.8%)
*Hereditary*	12 (9.4%)
*Interstitial*	11 (8.6%)
*Systemic*	8 (6.3%)
*Unknown/other*	27 (21.0%)
Diabetes mellitus	47 (36.7%)
Nonsmoker	40 (31.3%)
Log_2_ High-sensitivity troponin I	3.2 ± 1.4
Log_2_ NT-proBNP	8.5 ± 2.7
Log_2_ GDF-15	11.7 ± 1.1
LDL cholesterol (mg/dL)	93.7 ± 38.6
HDL cholesterol (mg/dL)	48.3 ± 16.4
Calcium (mg/dL)	9.1 ± 0.7
Phosphate (mg/dL)	4.1 ± 1.3
Log_2_ PTH	7.0 ± 1.4
25-hydroxyvitamin D (ng/mL)	20.8 ± 10.3
Left anterior descending artery calcification (AU) *	222.7 ± 382.8
Global coronary artery calcification (AU) *	543.6 ± 1250.6
Agatston score ≥ 400 *	39 (30.5%)

* Data available for 127 patients.

**Table 2:** Variables associated with significant global coronary artery calcification (Agatston score ≥ 400): univariable analysis.

Variable	Agatston Score < 400 (n = 88)	Agatston Score ≥ 400 (n = 39)	*p*
Age (years)	60 ± 7	62 ± 7	0.071
Male sex	44 (50.0%)	26 (67.0%)	0.082
BMI (kg/m^2^)	28.0 ± 5.4	29.0 ± 5.3	0.351
CKD stage 5	22 (25.0%)	17 (43.6%)	0.036
eGFR (mL/min/1.73 m^2^)	42.8 ± 31.1	28.2 ± 26.4	0.008
Diabetes mellitus	21 (23.9%)	26 (66.7%)	<0.001
Nonsmoker	24 (27.3%)	16 (41.0%)	0.124
Log_2_ high-sensitivity troponin I	2.9 ± 1.2	3.9 ± 1.4	<0.001
Log_2_ NT-proBNP	7.9 ± 2.4	9.8 ± 2.9	<0.001
Log_2_ GDF-15	11.4 ± 1.1	12.3 ± 0.9	<0.001
dp-ucMGP (pmol/L)	1520 ± 1222	1932 ± 1262	0.086
LDL cholesterol (mg/dL)	99.3 ± 41.4	81.5 ± 28.7	0.006
HDL cholesterol (mg/dL)	50.0 ± 17.4	44.6 ± 13.3	0.060
Calcium (mg/dL)	9.1 ± 0.7	9.1 ± 0.7	0.755
Phosphate (mg/dL)	3.8 ± 1.0	4.7 ± 1.7	0.004
Log_2_ PTH (pg/mL)	6.9 ± 1.3	7.2 ± 1.6	0.343
25-hydroxyvitamin D (ng/mL)	21.8 ± 9.4	18.9 ± 12.1	0.144

**Table 3:** Multivariable logistic regression models for severe coronary artery calcification (Agatston score ≥ 400).

Model	Variable	OR	95% CI	*p*	n
Primary	eGFR (per 1 mL/min/1.73 m^2^)	1.009	0.984–1.035	0.478	127
Primary	Diabetes mellitus	5.560	2.110–14.651	0.001	127
Primary	Log2 GDF-15 (per doubling)	2.105	1.045–4.240	0.037	127
Primary	LDL cholesterol (per 1 mg/dL)	0.997	0.983–1.010	0.621	127
Primary	Phosphate (per 1 mg/dL)	1.558	1.040–2.333	0.032	127
Primary + age and sex	Age (per year)	1.026	0.955–1.102	0.480	127
Primary + age and sex	Male sex	1.679	0.603–4.672	0.321	127
Primary + age and sex	eGFR (per 1 mL/min/1.73 m^2^)	1.010	0.984–1.036	0.461	127
Primary + age and sex	Diabetes mellitus	4.548	1.649–12.542	0.003	127
Primary + age and sex	Log2 GDF-15 (per doubling)	2.008	0.976–4.131	0.058	127
Primary + age and sex	LDL cholesterol (per 1 mg/dL)	0.998	0.984–1.012	0.757	127
Primary + age and sex	Phosphate (per 1 mg/dL)	1.682	1.081–2.617	0.021	127
Primary + hs-cTnI	eGFR (per 1 mL/min/1.73 m^2^)	1.014	0.988–1.040	0.295	127
Primary + hs-cTnI	Diabetes mellitus	5.596	2.048–15.291	0.001	127
Primary + hs-cTnI	Log2 GDF-15 (per doubling)	1.879	0.915–3.859	0.086	127
Primary + hs-cTnI	LDL cholesterol (per 1 mg/dL)	0.997	0.983–1.011	0.673	127
Primary + hs-cTnI	Phosphate (per 1 mg/dL)	1.522	1.005–2.304	0.047	127
Primary + hs-cTnI	Log2 hs-cTnI (per doubling)	1.515	1.045–2.195	0.028	127

**Table 4:** Pearson correlations between Log2-transformed GDF-15 and continuous variables and multivariable linear regression analysis.

Variable	r	*p*	Standardized β	95% CI for B	*p*
Age (years)	0.135	0.128	-	-	-
BMI (kg/m^2^)	−0.155	0.080	-	-	-
Diabetes mellitus	-	-	0.220	0.288–0.746	<0.001
eGFR (mL/min/1.73 m^2^)	−0.794	<0.001	−0.530	−0.025–−0.015	<0.001
Log_2_ High-sensitivity troponin I	0.405	<0.001	−0.034	−0.121–0.064	0.542
Log_2_ NT-proBNP	0.746	<0.001	0.375	0.096–0.220	<0.001
LDL cholesterol (mg/dL)	−0.250	0.004	−0.005	−0.003–0.003	0.916
HDL cholesterol (mg/dL)	−0.112	0.210	-	-	-
Calcium (mg/dL)	−0.342	<0.001	−0.044	−0.249–0.105	0.422
Phosphate (mg/dL)	0.427	<0.001	0.009	−0.087–0.103	0.868
Log_2_ PTH	0.502	<0.001	−0.022	−0.117–0.082	0.732
25-hydroxyvitamin D (ng/mL)	−0.156	0.079	-	-	-
Left anterior descending artery calcification (AU) *	0.313	<0.001	-	-	-
Global coronary artery calcification (AU) *	0.334	<0.001	-	-	-

* Data available for 127 patients.

**Table 5:** Univariable Cox regression analysis of variables associated with MACE.

Variable	HR	95% CI	*p*
Age (per year)	1.051	0.950–1.163	0.335
Male sex	0.216	0.045–1.043	0.056
BMI (per kg/m^2^)	1.075	0.949–1.217	0.254
CKD stage 5	8.469	1.756–40.834	0.008
eGFR (per mL/min/1.73 m^2^)	0.916	0.842–0.997	0.043
Diabetes mellitus	3.532	0.882–14.143	0.075
Smoker	2.796	0.750–10.415	0.125
Log_2_ High-sensitivity troponin I	1.198	0.780–1.840	0.408
Log_2_ NT-proBNP	1.632	1.249–2.132	<0.001
Log_2_ GDF-15	2.679	1.278–5.625	0.009
dp-ucMGP (per pmol/L)	1.000	1.000–1.001	0.367
LDL cholesterol (per mg/dL)	0.989	0.969–1.010	0.311
HDL cholesterol (per mg/dL)	0.908	0.836–0.987	0.024
Calcium (per mg/dL)	0.451	0.194–1.048	0.064
Phosphate (per mg/dL)	1.741	1.284–2.361	<0.001
Log_2_ PTH	1.928	1.195–3.112	0.007
25-hydroxyvitamin D (per ng/mL)	0.961	0.893–1.034	0.290
Left anterior descending artery calcification (per AU) *	1.002	1.001–1.003	<0.001
Global coronary artery calcification (per AU) *	1.001	1.001–1.001	<0.001
Agatston score ≥ 400 *	8.223	1.012–66.842	0.049

Note: * Data available for 127 patients.

## Data Availability

The raw data supporting the conclusions of this article will be made available by the authors on request.

## Supplementary Materials

The following supporting information can be downloaded at https://www.mdpi.com/article/10.3390/life16101661/s1, Table S1: Sensitivity analysis of the association between GDF-15 and severe coronary artery calcification restricted to participants not receiving dialysis (n = 95).

## Abbreviations

The following abbreviations are used in this manuscript:

AUC	area under the curve
AU	Agatston units
BMI	body mass index
CAC	coronary artery calcification
CI	confidence interval
CKD	chronic kidney disease
dp-ucMGP	dephosphorylated-uncarboxylated matrix Gla protein
eGFR	estimated glomerular filtration rate
ESKD	end-stage kidney disease
GDF-15	growth differentiation factor 15
HDL	high-density lipoprotein
HR	hazard ratio
LDL	low-density lipoprotein
MACE	major adverse cardiovascular events
MGP	matrix Gla protein
NT-proBNP	N-terminal pro-B-type natriuretic peptide
OR	odds ratio
PTH	parathyroid hormone
PTCA	percutaneous transluminal coronary angioplasty
ROC	receiver operating characteristic
SD	standard deviation
SHR	subdistribution hazard ratio
