# Supplementary material for: Growth Differentiation Factor 15 Is Associated with Severe Coronary Artery Calcification in Patients with Chronic Kidney Disease
Source: Life (Basel). 2026 Oct 2;16(10):1661. doi: 10.3390/life16101661 (PMC13705942; doi:10.3390/life16101661)
Supplement: Supplementary file 1 [file life-16-01661-s001.zip › life-4543643-supplementary.pdf]

Table S1. Sensitivity analysis of the association between GDF-15 and severe coronary artery calcification restricted to participants not receiving dialysis (n = 95).

Binary logistic regression models restricted to participants not receiving dialysis (n = 95). Model 1 included eGFR, diabetes mellitus, log2-transformed GDF-15, LDL cholesterol, and serum phosphate. Model 2 additionally included age and sex.

**Model**	**Variable**	**OR**	**95% CI**	**p**
Model 1	GDF-15 (per doubling)	2.597	0.932-7.233	0.068
	Diabetes mellitus	5.114	1.407-18.595	0.013
	Serum phosphate (per 1 mg/dL)	1.955	0.857-4.461	0.111
	LDL cholesterol (per 1 mg/dL)	0.998	0.983-1.013	0.792
	eGFR (per 1 mL/min/1.73 m^2)	1.019	0.989-1.050	0.209
Model 2	GDF-15 (per doubling)	3.066	0.975-9.637	0.055
	Diabetes mellitus	2.558	0.602-10.866	0.203
	Serum phosphate (per 1 mg/dL)	3.328	1.200-9.229	0.021
	LDL cholesterol (per 1 mg/dL)	1.004	0.987-1.021	0.648
	eGFR (per 1 mL/min/1.73 m^2)	1.031	0.995-1.068	0.088
	Male sex	8.725	1.777-42.827	0.008
	Age (per year)	1.049	0.945-1.164	0.373

Abbreviations: CI, confidence interval; eGFR, estimated glomerular filtration rate; GDF-15, growth differentiation factor 15; LDL, low-density lipoprotein; OR, odds ratio. GDF-15 was log2-transformed; therefore, the OR corresponds to a doubling of the original concentration.
